# Commentary: Perceptions of care quality and human rights in Italian outpatient facilities during and after the pandemic

**DOI:** 10.3389/fpubh.2026.1880634

**Published:** 2026-07-20

**Authors:** Viviane Kovess-Masfety, Alessandra Perra, Mauro Giovanni Carta

**Affiliations:** 1 LPPS, Paris Cité University, Paris, France; 2 Department of Medical Sciences and Public Health, University of Cagliari, Cagliari, Italy

**Keywords:** human rights, mental health, quality of care, QualityRights, wellbeing

The study by Cantone et al. (1) provides important evidence on the perception of care quality and human rights in Italian outpatient mental health services. In this commentary, we discuss the broader implications of their findings within the global framework of rights-based mental health care and highlight the role of scalable monitoring tools in sustaining attention to human rights in a context of growing geopolitical and financial constraints.

The article (1) demonstrates how a relatively simple and well-established international tool, the WellBeing at Work and Respect for Human Rights Questionnaire (WWRR), a validated and internationally applied instrument for assessing perceived respect for human rights in healthcare settings (2), can effectively measure perceived respect for human rights among both service users and healthcare professionals. This finding is particularly relevant in contemporary mental health systems, where the evaluation of rights-based outcomes remains a critical yet often underdeveloped dimension.

The WWRR tool is rooted in the World Health Organization's QualityRights initiative, one of the most comprehensive global efforts to promote a rights-based approach to mental health care. This framework integrates top-down and bottom-up strategies, combining policy reform with capacity-building among users, professionals, and advocacy groups (3–6). QualityRights has demonstrated that structured interventions can lead not only to increased awareness but also to measurable improvements in service organization and delivery. Notably, large-scale interventions have contributed to the transformation of mental health systems in settings such as Ghana and Gujarat, India (5, 7, 8). Furthermore, the initiative has strengthened methodological rigor in evaluating psychosocial interventions, promoting the adoption of evidence-based approaches, including randomized controlled trials (9, 10).

These developments must be interpreted within a broader global context. Recent United Nations and World Health Organization reports consistently highlight that human rights violations against persons with psychosocial disabilities remain widespread worldwide. Persistent issues include coercive practices, involuntary hospitalization, institutionalization, inadequate living conditions, and enduring stigma and discrimination (11, 12). Within this scenario, tools such as the WWRR assume a strategic role by enabling the systematic monitoring of rights-related outcomes in routine care settings.

However, current global dynamics pose significant challenges to the advancement of rights-based mental health systems. Increasing geopolitical tensions and rising military expenditures have been associated with reductions in international aid and decreasing investment in social and health policies (13). These trends risk limiting the implementation and sustainability of complex programs such as QualityRights, which require continuous institutional and financial support (14). Paradoxically, these constraints emerge at a time when the need to monitor and prevent human rights violations is likely to increase, particularly in fragile and crisis-affected contexts (15).

In this framework, the contribution of Cantone et al. (1) is particularly valuable. Their findings demonstrate that even in resource-constrained or politically challenging environments, relatively simple and scalable tools can maintain attention on human rights in mental health care. Beyond their immediate empirical implications, such approaches contribute to sustaining a culture of accountability and continuous evaluation, which is essential for preserving and advancing rights-based practices.

In conclusion, the study conveys a key message for public health: monitoring human rights in mental health care does not necessarily require complex infrastructures but can be effectively supported by standardized, feasible, and widely applicable tools. In a period marked by global uncertainty and competing priorities, maintaining the systematic monitoring of human rights in mental health care is not only a methodological necessity but a fundamental safeguard against regression in the protection of vulnerable populations.
